# The Coming Meeting of the Texas State Medical Association—Medical Legislation and the Subject of a Charter

**Published:** 1889-01

**Authors:** 


F. E. DANIEL, M. D., EDITOR AND PUBLISHER.

THIS JOURNAL, by unanimous vote of the members, was made the Official Organ of the Austin District Medical Society.

COLLABORATORS:

*Dr. R. M. Swearingen, Austin.*

*Prof. B. E. Hadra, M. D., Galveston.*

*Prof. Geo. Cupples, M. D., San Antonio.*

*Dr. T. C. Osborn, Cleburne, Texas.*

*Dr E. J. Doering, Chicago.*

*Dr. E. J. Beall, Fort Worth.*

*Dr Odo Betz, Germany.*

*Dr. J. W. McLaughlin, Austin.*

*Dr. T. J. Tyner, Austin.*

*Prof. J. F. Y. Paine, M. D., Galveston.*

*Dr. R. H. L. Bibb, Mexico.*

*Dr. T. J. Bennett. Austin.*

*Dr. Bat Smith, Wharton.*

*Dr. E. Meierhof, New York.*

*Prof. W. B. Rogers, M. D., Memphis, Tenn.*

### THE COMING MEETING OF THE TEXAS STATE MEDICAL ASSOCIATION—MEDICAL LEGISLATION AND THE SUBJECT OF A CHARTER.

Let it be borne in mind that the Texas State Medical Association will meet in the beautiful and historic city of San Antonio, on the fourth Tuesday in April next,—only a few months hence, —and let there be a larger attendance than for several years past.

San Antonio is easy of access from any point in Texas, and the railroads having gotten over their little *pique* at the doctors, have begun again to do the handsome thing by giving them special rates to and from their conventions.

The attractions will be, if possible, even greater than they were at Galveston, socially and scientifically. The San Antonians are noted for their generous hospitality, are proud of their "Alamo City," and will do all in their power to make their guests happy. Business of great importance will come up for discussion; a new constitution and by-laws are to be adopted, among other things, and the subject of medical legislation is to be thoroughly considered. With the narrow-minded men of the "Bell-of-Cook" sort relegated to the shades of private life, and a better class of men elected to the legislature, it is hoped that the subject will receive more respectful consideration at the hands of the law-makers when again presented by the organized medical profession, than ever heretofore.

The subject of chartering the State Medical Association should, and probably will, come up for discussion. In our judgment, this is the most imporant topic that could receive the attention of that body; the step has become, in our opinion, one of most urgent necessity. We bear well in mind the summary manner in which the proposal to charter the Association was "sat down on" at Belton in '84, but that fact should not deter us from reviving the question. Perhaps at that time there was no real necessity of such a course, but at the present time there can be no question as to its advisability; the *status* of medical matters is much changed since then, and this change has necessitated something of the sort. Then, there were perhaps, not half a dozen local medical organizations in the State; now there are one or more in nearly every organized county; and not only is the supremacy of the State Association, and its right to tribute, (of representation, allegiance and money), from these organizations very questionable, but its very integrity is threatened by their advent. What is to hinder—should such a step be thought proper—any or all of the District Societies declaring themselves independent of the State Association, and all sufficient for the needs of the profession in their respective sections? The rapid growth of these larger bodies, and their popularity, render them, in a sense, rivals of the State Association. A member may say, "we derive as much benefit from our local society meetings as we do from those of the State Association. We do not have so far to go, nor so much to pay. We have not time to attend other meetings; I believe I will resign from the State Association." And this can be said with much truth. Either the State Association must adopt some plan whereby all local societies can be kept in the leading strings, or it must make itself indispensable to members. It is a fact, that while there are represented on the roll of the State Association some twenty odd auxiliary associations, there are important organizations which are not on the roll at all, and at least one which we recall, with a membership of fifty, which, by vote, refused to send representation to the yearly convention of the State Association. A member may reasonably say, "why should I pay dues in so many organizations when my District or County Society is sufficient for my time and wants?" One cannot take all the journals, and selects that one, or one or two which best answer his requirements. So it is with the associations. There is no inherent right in the State Association to be considered superior to any District Association; but if a County or District Association could only acquire authority to organize—by procuring a charter from the State Association—it would be different, and organizations in medicine, upon the only proper basis, could be accomplished forthwith, and the permanency and integrity of the State Association would be guaranteed, the profession consolidated and strengthened.

Let our committee on legislation ponder these suggestions, and be prepared to report upon the subject of applying to the State for a charter.

We would be glad to hear from members on the subject—would like to get at a concensus of opinion prior to the meeting.

With good crops, and consequently more money in Texas, it is hoped that the attendance at San Antonio will be large. The time is coming, and it will soon be here, when not to belong to the organized body of the profession of Texas, will be to be a marked man, and the presumption will be that because one is not a member, one cannot be a member.

Organization is abroad; all who are entitled, by virtue of proper professional and moral standing, to become members, should do so. There are many who can never hope to be taken into the fold, and those physicians who have a proper professional pride should be careful not to be thought one of these.

